# Comparative Analysis of Strength of Differently Activated Denture Base Materials Including Recent Acetal Resin-Based Biodentaplast

**DOI:** 10.7759/cureus.54676

**Published:** 2024-02-22

**Authors:** Shakir Ahmed R, Bharanija Kalidasan Selvi, Shyam Sundar Shankar, Annapoorni Hariharan

**Affiliations:** 1 Prosthodontics, Sri Ramachandra Institute of Higher Education and Research, Chennai, IND; 2 Prosthodontics, Meenakshi Ammal Dental College and Hospital, Chennai, IND

**Keywords:** bio-dentaplast, flexible dentures, injection molding, acetal resin, denture base resin, denture base

## Abstract

Aim and objectives: The aim of this study is to comparatively analyse the compressive and tensile strength of different types of record base materials made of different materials and processing techniques.

Materials and methodology: The compressive and tensile strength of 4 types of injection moulded materials were compared with a control of conventional compression moulded material. Twenty test specimens (10 tensile and 10 compressive) were fabricated from each material. A test was done using the Instron 3382 (Norwood, MA, USA) universal testing machine.

Results: Compressive and tensile test values showed significant differences between the record base resin groups tested for both compressive and tensile strength tests (p=0.00). The mean tensile strength value was greatest for Group V (66.0 MPa) and lowest for Group III (41.9MPa) and the mean compressive strength value was greatest for Group I (74.5 MPa) followed by Group V (70.2 MPa) and lowest for Group III (10.8 MPa).

Conclusion: Injection moulded acetal resin showed the highest tensile strength value; it was comparable to that of conventional compression moulded polymethyl methacrylate (PMMA). Compression moulding is reported to have the highest compressive strength values followed by injection moulded acetal resin material. Injection moulded acetal resin material attributed to its advantages and superior strength value, can be used as a material of choice in various clinical scenarios.

## Introduction

Since time immemorial, dentures have been a common modality of restoration in prosthodontics. Prosthetic resins are used widely for fabricating prostheses, including complete or removable partial dentures, transitional prostheses, and implant-supported prostheses [1]. Conventional heat polymerized polymethyl methacrylate (PMMA) denture base resin has been in use since the 1940s due to its good biocompatibility, colour stability and minimal water sorption. However, it involves a tedious and time-consuming laboratory procedure and undergoes polymerization shrinkage. In order to overcome these shortcomings as well as to improve its applications, various modifications to its composition and techniques have been tried; additionally, various newer materials have also been introduced. The injection moulding technique of heat processing minimizes the requirements of a conventional flask; it is advantageous as it compensates for dimensional change as well as polymerization shrinkage since it uses fluid resin [2]. The evolution of thermoplastic/polyamide resin led to a more commercially attractive denture base material due to its better flexibility and improved patient comfort. Flexible resins in removable partial dentures eliminate the need for a metal framework reviving the success of removable partial dentures [3]. The development of polymer chemistry produced alternative materials such as acetal resins (polyoxymethylene-based materials) and ether-based PEEK (polyetheretherketone) to replace PMMA-based resins which possess properties such as good strength and biocompatibility as well as metal alloys with high strength and thermal conductivity used in frameworks to enhance esthetics, physical properties as well as patient acceptance [4]. This study comparatively evaluates the compressive and tensile strength of conventional compression moulded PMMA and more recent materials processed by an injection moulding technique.

## Materials and methods

A total of 100 samples were to be fabricated, 20 specimens of each material (n=20) out of which 10 samples were to be tested for compressive load (n=10) and 10 samples for tensile load (n=10). Materials used and their description are available in Table 1.

**Table 1:** Description of various groups of materials used PMMA: Polymethyl methacrylate

Group	Commercial name	Type of material	Type of processing	Availability	Processing description
I	Meliodent (Hareus Kulzer)	PMMA	Compression moulding	Polymer-Monomer Ratio: 23.4 g/10ml	20 mins at 70-degree Celsius, 25 mins in boiling water
II	SR-Ivocap HI (Ivoclar Vivadent)	PMMA	Injection moulding	Polymer-Monomer Ratio: 20 g/30ml	Injected with pressure of 6 bars and placed in boiling water for 35 mins
III	Valplast (Valplast Corp., USA)	Polyamide	Injection moulding	Single Pellet	Preheated for 8 mins at 248-265^o^C Injected at 5 bars pressure for 3 mins
IV	Breflex (Bredent, Germany)	Polyamide	Injection moulding	Single Pellet	Preheated for 8 mins at 222^o^C and injected at 5 bars pressure
V	BioDentaplast (Bredent, Germany)	Acetal resin	Injection moulding	Single Pellet	Preheated for 15 mins at 220^o^C, injected at 7.2 bars pressure for 25 seconds

**Figure 1:**
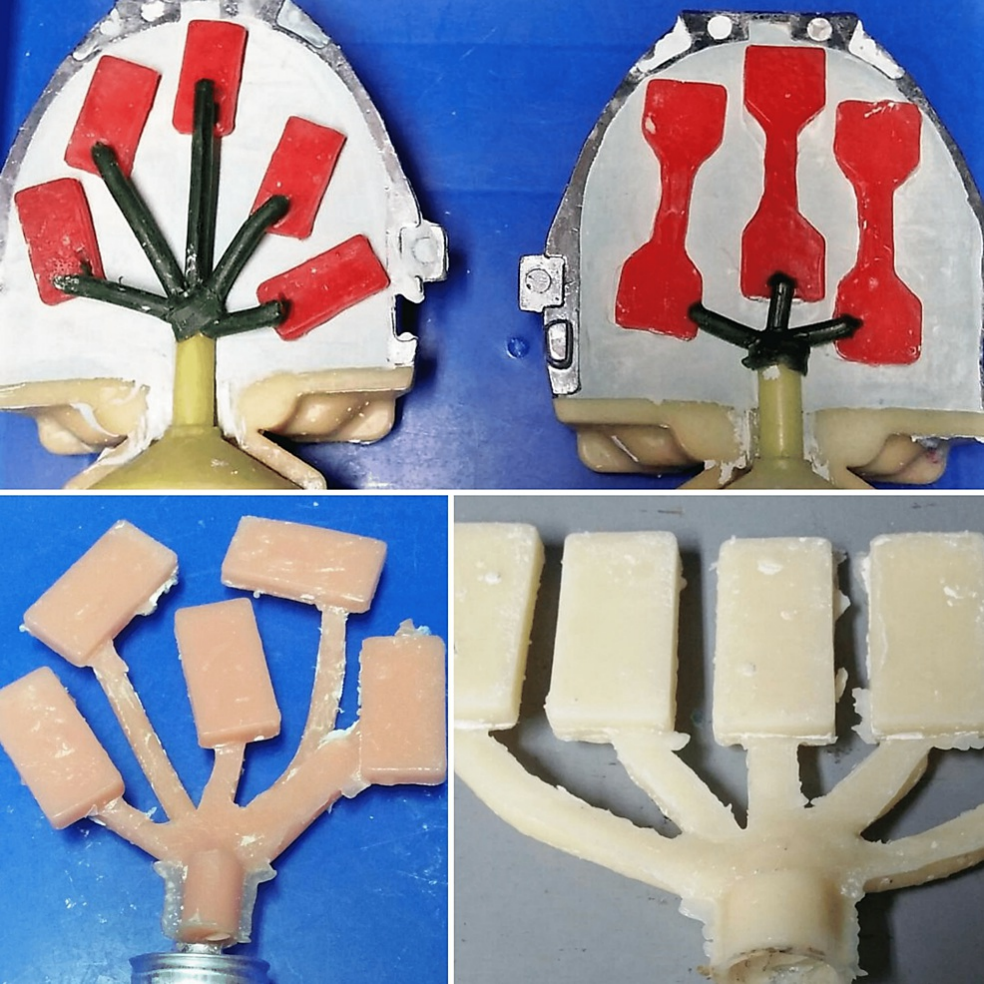
Fabrication of test samples

**Figure 2:**
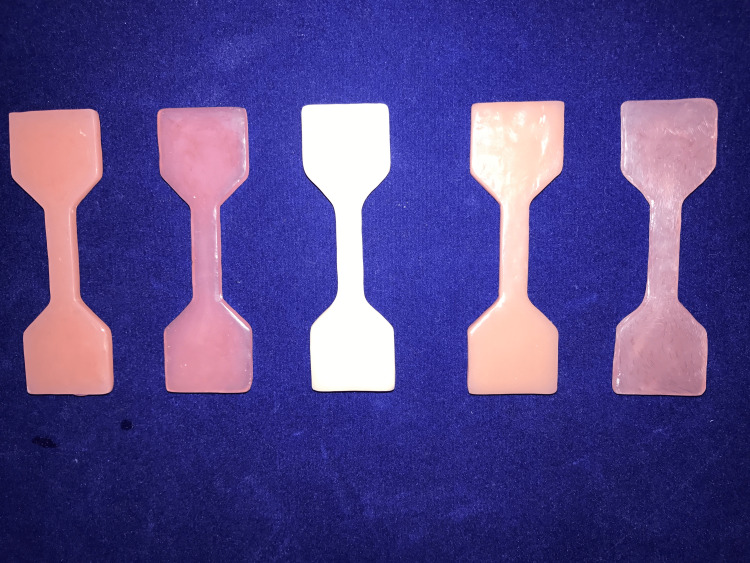
Tensile strength samples

**Figure 3:**
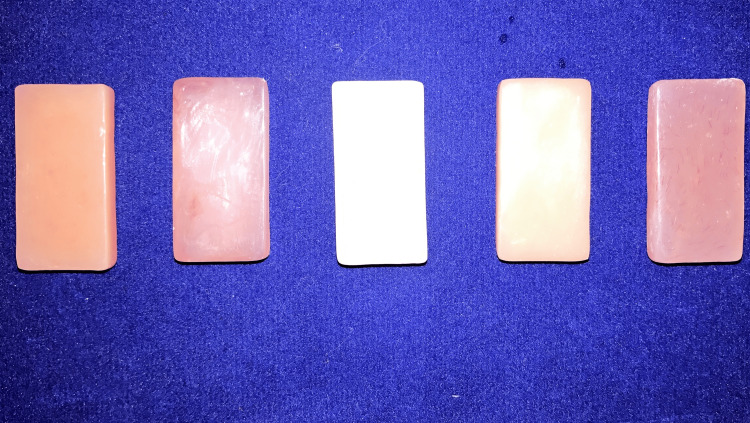
Compressive strength samples

Wax patterns were fabricated by pouring molten wax into the hollow cavity in the milled-metal mould with dimensions in accordance with the use of the universal testing machine. For tensile strength testing, a dumb-bell-shaped specimen measuring 65 mm in length, 3 mm in thickness and 20 mm in breadth at both holding ends and 6.5 mm at the centre is to be made. For compressive strength testing, a rectangular block specimen measuring 30 mm in length, 15 mm in breadth and 3 mm in thickness is to be made. Samples were then fabricated using two techniques, compression moulding and injection moulding (Figure 1-3).

**Figure 4:**
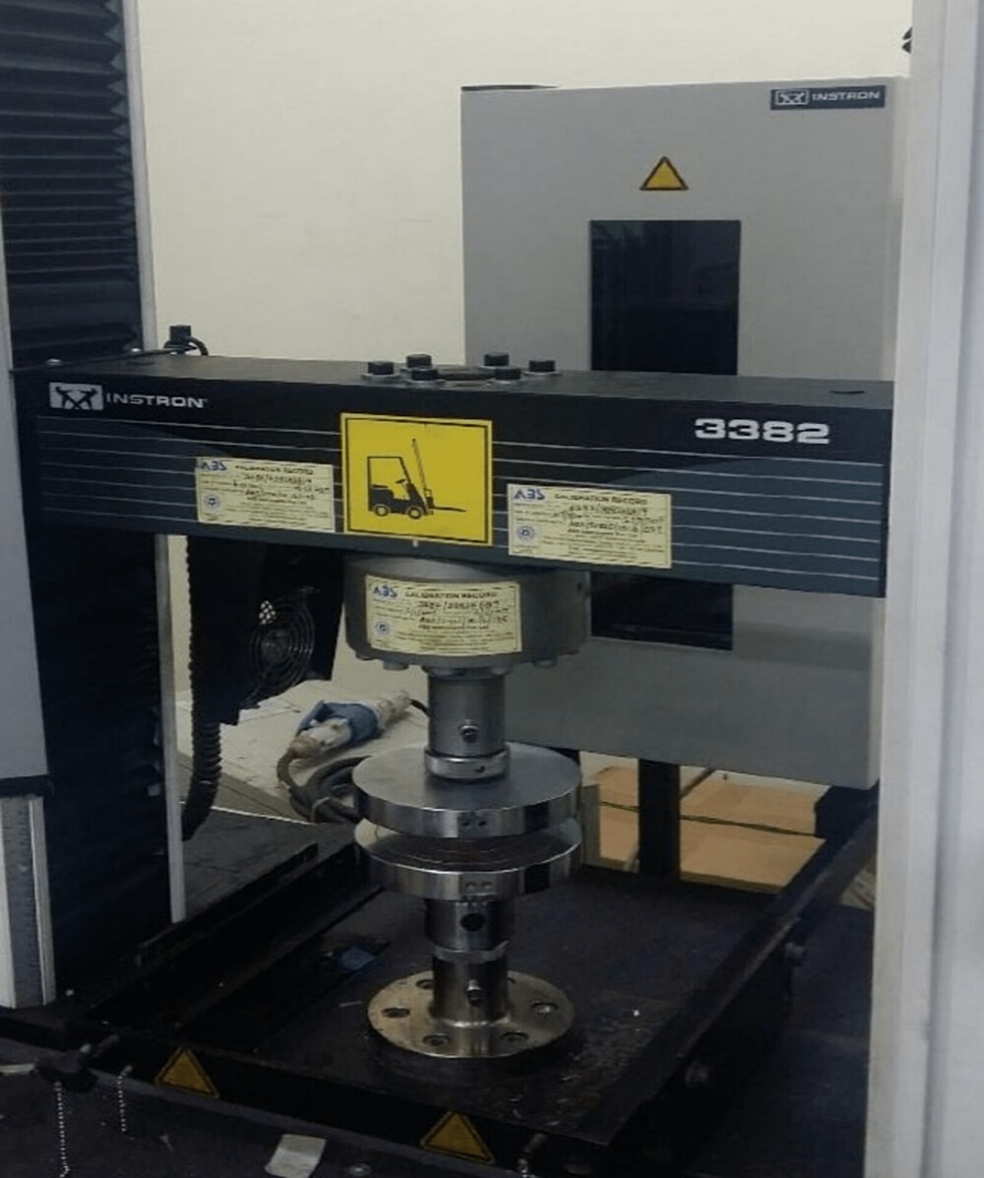
Testing for compressive strength

**Figure 5:**
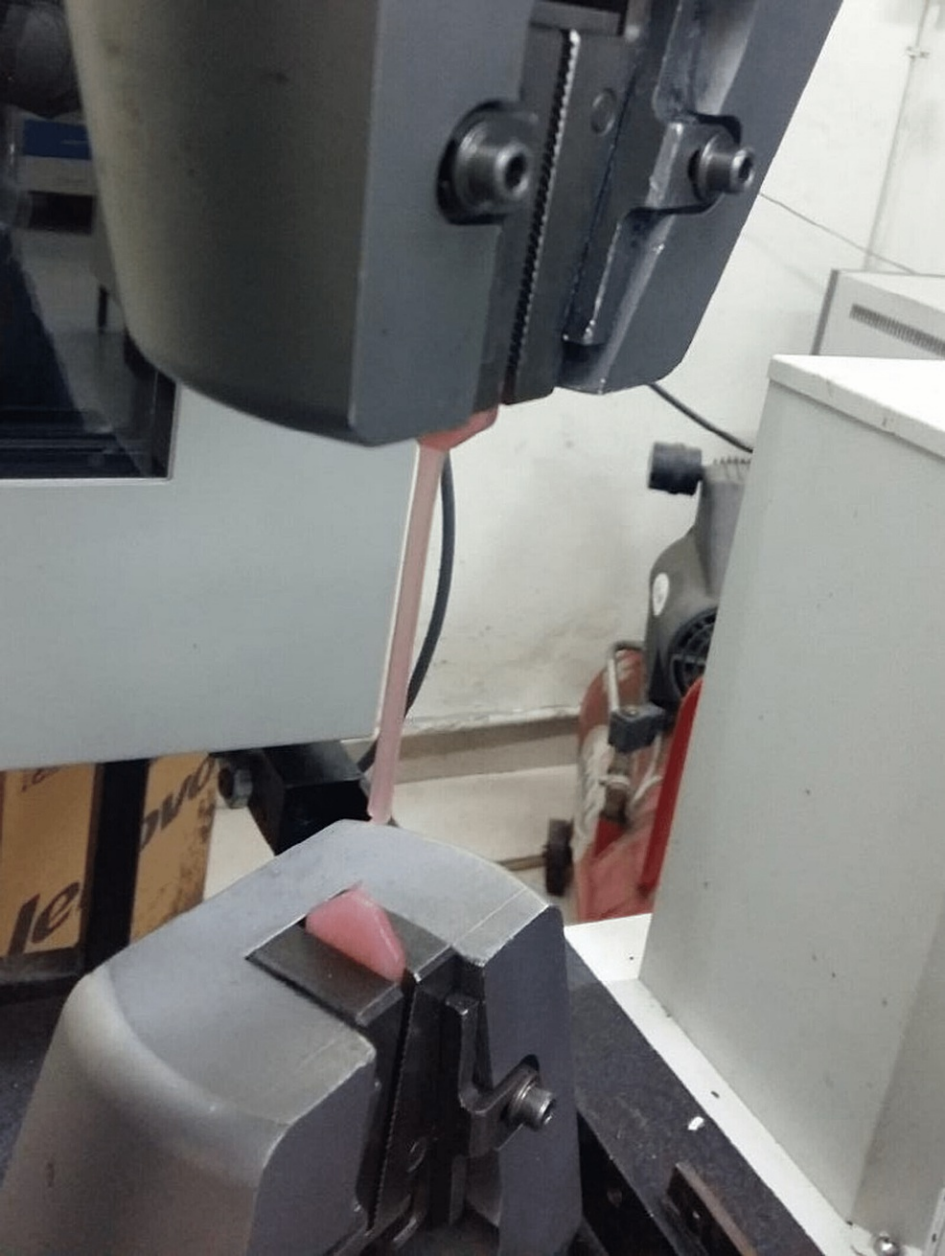
Testing for tensile strength

The universal testing machine Instron 3382 (Norwood, MA, USA) was used to test both compressive and tensile strength values; the testing steps and machine settings were followed according to manufacturer guidelines and the testing of samples performed (Figure 4, 5).

## Results

The values obtained for compressive strength (Table 2) and tensile strength (Table 3) of the test groups were tabulated.

**Table 2:** Compressive strength values obtained of various material samples (in MPa) PMMA: Polymethyl methacrylate

Group I (Control)	Group II (Injection moulded PMMA)	Group III (Polyamide-I-Valplast)	Group IV (Polyamide-II-Breflex)	Group V (Acetal resin)
77.65	55.64	10.60	21.52	64.68
72.98	56.35	12.86	26.32	65.37
74.12	45.85	9.00	26.55	78.38
72.32	48.75	11.24	24.66	72.59
77.78	51.60	10.23	23.72	67.27
74.05	47.79	12.32	25.84	69.37
75.10	53.45	11.45	22.98	68.69
75.45	49.64	10.86	24.43	78.21
73.56	54.32	9.72	25.93	73.43
72.62	52.90	10.34	23.46	64.59

**Table 3:** Tensile strength values obtained of various material samples (in MPa) PMMA: Polymethyl methacrylate

Group I (Control)	Group II (Injection moulded PMMA)	Group III (Polyamide-I-Valplast)	Group IV (Polyamide-II-Breflex)	Group V (Acetal resin)
60.63	51.83	39.87	46.00	73.36
56.62	46.29	40.45	45.82	68.32
60.69	56.55	42.99	42.37	61.36
56.55	51.63	43.33	48.39	65.12
54.79	54.53	43.32	46.55	62.12
59.62	49.39	41.43	45.78	66.73
57.69	52.64	43.64	44.62	67.15
54.63	53.55	40.74	42.76	64.42
60.62	55.42	39.52	48.14	62.57
56.65	49.23	43.71	47.01	68.85

The obtained data (Table 2, 3) was statistically analysed using the statistical software Statistical Package for Social Sciences (SPSS), version 21.0 (IBM Corp., Armonk, NY) (Table 4). The Shapiro-Wilk normality test was performed.

**Table 4:** Descriptive analysis of the data obtained

		N	Mean	SD	Min	Max
Tensile strength	Control	10	57.8490	2.38048	54.63	60.69
	Injection moulded	10	52.1060	3.14032	46.29	56.55
	Valplast	10	41.9000	1.66691	39.52	43.71
	Breflex	10	45.7440	2.01711	42.37	48.39
	Bio-D	10	66.0000	3.66893	61.36	73.36
	Total	50	52.7198	9.04673	39.52	73.36
Compressive strength	Control	10	74.5630	1.93596	72.32	77.78
	Injection moulded	10	51.6290	3.51077	45.85	56.35
	Valplast	10	10.8620	1.16037	9.00	12.86
	Breflex	10	24.5510	1.64103	21.52	26.55
	Bio-D	10	70.2580	5.20693	64.59	78.38
	Total	50	46.3726	25.44513	9.00	78.38

Following the above an ANOVA test was performed to analyse the compressive and tensile strength data and it was concluded that the p value was significant (<0.001) (Table 5). Tukey HSD (Honest Significance Difference) test was applied for comparison of different groups.

**Table 5:** Analysis of variance (ANOVA) test results

		Sum of squares	Df	Mean square	F	Sig.
Tensile strength	Between groups	3687.790	4	921.948	128.632	.000
	Within groups	322.530	45	7.167		
	Total	4010.320	49			
Compressive strength	Between groups	31300.257	4	7825.064	828.488	.000
	Within groups	425.025	45	9.445		
	Total	31725.281	49			

## Discussion

To investigate the effectiveness of the Injection moulding system or technique of denture processing as an alternative to conventional compression moulding processing system, the mechanical properties of different denture base materials processed using the Injection moulding processing method were tested. Though transverse strength represents the type of loading in dentures more closely than tensile strength and compressive strength, lack of knowledge and lesser number of previous literature on tensile and compressive testing of denture base materials is the reason for us to test these properties in our study.

Tensile strength

Acetal resin-based BioDentaplast (Bredent, Germany) was found to be most resistant to tensile load (66.0 MPa) among the groups tested. PMMA-based resins Meliodent (Hareus Kulzer) (57.8 MPa) and SR-Ivocap HI (Ivoclar Vivadent) (41.9 MPa) showed comparatively less resistance to tensile load but the values obtained were 20% greater than the values for polyamide-based Valplast (Valplast Corp., USA) (41.9 MPa) and Breflex (Bredent, Germany) (45.7 MPa) materials. Of the 5 groups tested, Valplast showed the lowest values of tensile strength (Figure 5). Mean tensile strength values of conventional compression moulded PMMA (Meliodent) of 57.8 Mpa were lesser in comparison to those values in previous literature by Sehajpal et al. (61.5 MPa), Bortun et al. (65.8 MPa), Ghiban et al. (64 MPa) and more in comparison to values in study by Al-Wahab et al. (51.2 Mpa) [5-8].

Compressive strength

Conventional compression moulded PMMA (Meliodent) showed the highest compressive strength values (74.5MPa). Acetal resin-based BioDentaplast (70.2 MPa) showed comparative values of compressive strength followed by injection moulded PMMA (SR-Ivocap HI - 51.6 MPa). Polyamide-based injection moulded resins showed lesser compressive strength values (Breflex - 24.5 MPa) of which Valplast (10.8 MPa) was the least (Figure 5). The compressive strength of conventional compression moulded PMMA denture base material (Meliodent - 74.5 MPa) was comparable to that in previous literature by Hashem et al. of 71.9 Mpa [9]. From testing for tensile and compressive strength, it was found that conventional compression moulded showed better values in terms of both mean compressive (74.5 MPa) and tensile strength (57.8MPa) than injection moulded PMMA (mean compressive = 51.6Mpa and mean tensile = 52.1MPa).

Polyamide-based thermoplastic denture base materials, unlike acrylic dentures, are made from a thermoplastic nylon resin that is ultrathin, very flexible and transparent and thus the esthetics are far superior to conventional acrylic/metal partial dentures. It allows for the fabrication of metal-free clasps or removable partial dentures. They are difficult to finish and polish. Though flexible, these polyamide-based materials require a certain level of strength to fulfil the specifications of denture-based materials. In our study, the polyamide-based Valplast and Breflex materials showed lower strength values compared to other materials. The mean compressive strength (45.7MPa) and tensile strength (24.5MPa) of Breflex polyamide material were better than that of Valplast (compressive = 10.8 MPa and tensile = 41.9Mpa).

Biodentaplast is a techno-polymer made from acetal (polyoxymethylene) resin. The polymerization of formaldehyde produces Acetal resins. An oxygen molecule connects a chain of alternating methyl groups. It is recommended for use as a clasp in removable partial dentures because of its various advantages, including wear resistance [10], superior abrasion resistance, and higher stiffness. It has a low water absorption rate, making it an ideal material for making interim surgical prostheses for larger oral defects. In view of better flexibility in comparison with cobalt-chromium clasps, the retentive clasp arm of acetyl resin engages deeper undercuts on the abutments [11]. In our study, BioDentaplast (Group V) showed the highest tensile strength (66.0 MPa) among all the groups tested and compressive strength value (70.2 MPa) only next to conventional compression moulded Meliodent (74.5 MPa). Wettability with saliva is an important factor that aids in denture base retention. Farcasiu and Pauma noted BioDentaplast to have superior wettability in comparison to other heat-cured acrylic resins tested [12]. This serves as an indication for the use of BioDentaplast as a material of choice for patients with decreased salivary flow or Xerostomia [12]. Acetal resin (BioDentaplast) frameworks may be as thin as 0.3-0.5 mm without any macroscopic defects so that the material is suitable for removable partial dentures with flexible, esthetic clasps [4]. Acetal resin can be used as an alternative material to conventional heat-cured acrylic resin [10].

Limitations

Determination of the flexural strength of the denture base materials would have contributed to better clinical relevance. Understanding the application of acetal resin-based clasps for removable partial dental prostheses and its efficiency requires for further clinical studies.

## Conclusions

Injection moulded acetal resin showed the highest tensile strength values; it was comparable to that of conventional compression moulded polymethyl methacrylate (PMMA). Compression moulded is reported to have the highest compressive strength values followed by the injection moulded acetal resin material. Between the two PMMA-based resins tested, compression moulded showed better properties than injection moulded denture material. Polyamide (nylon-based) materials recorded inferior compressive and tensile strength values in comparison with other types of resins (PMMA and acetal resin). Based on our study, it can be suggested that conventional compression moulded and injection moulded PMMA-based denture base resins still remain the material of choice for the fabrication of dentures. Acetal resin-based denture material BioDentaplast also shows comparable strength properties; additionally, its application as a removable partial denture framework material and as an esthetic tooth-coloured clasp enables its selection as a material for the fabrication of removable partial dentures.
